# A Hybrid Model for Leaf Diseases Classification Based on the Modified Deep Transfer Learning and Ensemble Approach for Agricultural AIoT-Based Monitoring

**DOI:** 10.1155/2022/6504616

**Published:** 2022-04-05

**Authors:** Maryam Saberi Anari

**Affiliations:** Department of Computer Engineering, Technical and Vocational University (TVU), Tehran, Iran

## Abstract

As possible diseases develop on plant leaves, classification is constantly hampered by obstacles such as overfitting and low accuracy. To distinguish healthy products from defective ones, the agricultural industry requires precise and error-free analysis. Deep convolutional neural networks are an efficient model of autonomous feature extraction that has been shown to be fairly effective for detection and classification tasks. However, deep convolutional neural networks often require a large amount of training data, cannot be translated, and need a number of parameters to be specified and tweaked. This paper proposes a highly effective structure that can be applied to classifying multiple leaf diseases of plants and fruits during the feature extraction step. It uses a deep transfer learning model that has been modified to serve this purpose. In summary, we use model engineering (ME) to extract features. Multiple support vector machine (SVM) models are employed to enhance feature discrimination and processing speed. The kernel parameters of the radial basis function (RBF) are determined based on the selected model in the training step. PlantVillage and UCI datasets were used to analyze six leaf image sets containing healthy and diseased leaves of apple, corn, cotton, grape, pepper, and rice. The classification process resulted in approximately 90,000 images. During the experimental implementation phase, the results show the potential of a powerful model in classification operations, which will be beneficial for a variety of future leaf disease diagnostic applications for the agricultural industry.

## 1. Introduction

Before they reach maturity, the diseases in the leaves of fruits, citrus, wheat, and rice can have a significant impact on their yields [1]. This necessitates rapid and precise diagnosis of leaf diseases in fruits, citrus, wheat, and rice as well as early delivery of tailored cure [2]. Human investigation-based recognition of leaf diseases is severely hampered due to the huge area, and a model capable of tackling this issue is urgently required. The plant itself typically has its own recognition model when a leaf disease of a certain sort is recognized. A problem with storage capacity will occur if each disease of fruits, citrus, wheat, and rice has its own recognition model. Multitask learning allows features to be shared and aided by one another. This allows the leaf disease recognition models to meet current needs while reducing storage space and increasing recognition accuracy. For this reason, a model that can detect and identify leaf diseases in fruits, citrus, wheat, and rice must be developed.

For image classification, the adoption of deep learning (DL) has been driven by recent advancements in computer vision and artificial intelligence [3–5]. Convolutional neural networks (CNNs) are an efficient model of DL structure. Self-learning, adaptability, and generalization are the hallmarks of CNN. The leaf diseases in fruits, citrus, wheat, and rice have been classified by machine vision approaches in numerous studies. Azadbakht et al. [6] achieved 82 percent correct classification of the normal and diseased rice leaves. Azadbakht et al. [6] employed machine learning (ML) approaches to analyze the wheat leaf hyperspectral data. Plant leaves are usually the first place where plant diseases are classified, and signs of most diseases may arise on the leaves [7, 8]. CNN requires no manual feature extraction as in classic machine vision (MV) procedures, which rely on manual classification. Instead, CNN only requires visual input. In recent years, deep learning approaches, specifically CNNs, have become the preferred method to overcome learning challenges [9, 10].

The network's ability to self-learn allows it to complete the image classification process successfully [11]. It has become increasingly common in recent years to use DL image classification strategy in agriculture for detecting plant diseases [12–15].

Three typical rice leaf diseases were categorized using a DL network that was trained using DenseNet and Inception models with ImageNet pretrained. The accuracy percentage was found to be 94.07 percent [16].

Support vector machines (SVM) and DL were utilized by Jiang et al. [17] to identify four distinct rice leaf diseases. Using SVM and CNN, they were able to extract the features from various leaf images and recognize the extracted features, with an accuracy of 96%. Transfer learning-based powdery mildew disease detection was proposed by Shin et al. [18]. Using six different models, they compared their categorization findings. In this case, they found that ResNet-50 had satisfactory accuracy in identifying objects, while AlexNet had the quickest recognition time. In order to diagnose grapevine leafroll disease, Gao et al. [19] employed ML techniques to analyze hyperspectral images of grape leaves. The disease in grapevine leafroll can be diagnosed in its asymptomatic stage using this model, which achieved an accuracy of 89.93 percent [19]. DL networks were utilized by Long et al. [20] to recognize four different forms of camellia diseases based on image processing analysis. They employed ImageNet to train AlexNet and then built a novel fully linked layer with an accuracy of 91.25%. The pretrained model can greatly enhance accuracy when there are few samples available. Generally, we can utilize the same machine learning model to do many tasks using multitask learning, which is a common practice. For the strawberry Verticillium wilt classification, researchers applied the multitask learning network of the attention system. Additionally, 99.95% of the time, they properly diagnosed strawberry Verticillium wilt [21]. Plant phenotyping was studied by Dobrescu et al. [22] using multitask learning. Genotype classification, leaf counting, and PLA estimation were achieved using the ResNet-50 model trained on ImageNet. Each of the three locations has a significant advantage over the other options.

To simulate realistic situations of leaf disease, Arsenovic et al. [23] used images taken in a range of weather conditions, at various angles and at different times of day, all with varying backdrops. In their study, the authors propose a novel two-stage neural network (NN) approach for classifying plant diseases in the context of a real environment. Their model's accuracy was 93.67%.

Karthik et al. [24] presented a design based on residual learning to classify significant aspects for detecting infection in tomato leaves. Their proposed approach makes use of the PlantVillage dataset, which contains three diseases, and CNN-learned features. They achieved 98 percent accuracy on the validation sets after fivefold cross-validation.

Sharma et al. [25] developed an approach for CNN model training. When tested on unlabeled data, the S-CNN model trained employing segmented images outperformed the F-CNN model by more than doubling its performance to 98.6% accuracy.

Sambasivam and Opiyo [26] suggested an approach for detecting cassava leaf disease in their study. They achieved an accuracy score of over 93% by using deep CNN built from scratch, class weight, and the Synthetic Minority Oversampling Technique.

Ma et al. [27] claimed that they used VGG to identify and classify four diseases in cucumber leaf leaves which resulted in crop loss. The classification was done on a vast volume of plant leaf images with the goal of classifying the presence or causes of disease in the leaves.

Too et al. [28] used four transfer learning (TL) models to classify the absence and presence of disease in plant leafs. These components included the VGG16, early V4, ResNet, and DenseNet structures. DenseNet, when compared to the other forms, generates more relevant responses with less processing time.

He also evaluated 12 different species of plants in another study [29], which were used to classify the presence or absence of disease in plants.

Kaya et al. [30] investigated datasets such as Flavia, Swedish, and UCI Leaf using TL for deep NNs in plant classification procedures. Similarly, Singh et al. [31] investigated the leaves of the corn plant using a deep NN model and suggested an automatic classification approach.

The study by Dhivyaa et al. [32] selected the appropriate features based on bidirectional long short-term memory (Bi-LSTM) to detect plant diseases by utilizing an expanded complexity network and other dense blocks. Data from cassava disease and PlantVillage dataset have demonstrated the validity of their proposed model. According to their findings, the proposed model for the diagnosis of cassava leaf disease achieved a maximum F1 score of 95.49%.

In a study by Bhujel et al. [33], the performance of the models was improved by using a lightweight convolution neural network with several attention modules. They were able to train their models with data about tomato leaf diseases. They evaluated the models' performance (F1 score, accuracy, and recall) according to standard classification accuracy metrics. With an average accuracy of 99.69%, the convolution block focus module was the most accurate.

By using R-CNN Mask, Storey et al. [34] segmented samples to diagnose leaf and rust disease in apple orchards. For object detection, segmentation, and sickness detection, three R-CNN Mask backbones were trained and evaluated. Using the R-CNN Mask model and the ResNet-50 backbone, they were able to detect extremely small rust-covered objects.

Prabu and Chelliah [35] have developed a new method of detecting mango leaf diseases. Over 388 images of healthy and ill subjects (mango anthracnose, soot mold, and bacterial black spot) were selected. MobileNetV2 is used during the learning phase, and the SVM is used to classify diseases at the end.

Singh et al. [36] used the AlexNet model to identify leaf disease in maize quickly and accurately. The results were verified with PlantVillage data. *Cercospora* and Gray are two common rust-based infections covered with 99.16% accuracy in the data collection.

A lightweight convolutional neural network, RegNet, was presented by Li et al. [37] for the detection of apple leaf disease. They were able to identify five different apple leaf diseases from 2141 images of healthy and diseased apple leaves. It achieved an overall accuracy of 99.23% in the test set and 99.8% in the validation set, with a learning rate of 0.0001.

Shahoveisi et al. [38] used ML techniques to model the risk of *Sclerotinia sclerotiorum*-induced disease development in canola and dry beans. Using a broad genome correlation study [39], they previously examined the genomic regions associated with *Leptosphaeria maculans* resistance in rapeseed.

Plant leaf diseases may be consistently diagnosed using deep learning. However, TL may be used to overcome the difficulty of small datasets in plant leaf disease recognition and significantly increase the approach's accuracy. A single structure can be utilized to perform multiple tasks through ensemble learning. To solve the challenge of recognizing leaf illnesses on two different plants, the study provides research on deep transfer learning and alternate learning. Improvements were made to the residual neural network (ResNet) model, while keeping the ResNet model's convolutional layer structure. As a result, these two components of the ResNet model's classification layer can be shared across different datasets. A new fully connected layer is built to address the various classification challenges that arise while working with several data sources. The loss function and optimizer for each classifier are unique. The convolutional layer of the ResNet is fixed during formal training, allowing the TL architecture to be used for transfer learning. Formal instruction utilizes only the newly developed categories and layers that are completely interconnected. Digital cameras may be used to take on-the-spot images utilizing fruits, citrus, and rice leaves and models to diagnose leaf diseases, allowing crops to be saved before irreparable harm occurs. Although several approaches exist for diagnosing fruit leaf disease, key questions remain unresolved.

1. Identifying and collecting critical information from fruit leaves is fairly difficult, as is distinguishing the features of particular diseases utilizing conventional image processing methods.
2. Due to the huge variability in the features of different diseases, the attributes of disease patterns must be thoroughly studied using a diverse set of images in a smart fashion.
3. The efficiency of ML techniques is entirely determined on the type of the hand-crafted features. As a result, feature extraction must be performed automatically in order to pick and learn the optimal collection of features for recognition.
4. Certain deep learning models make advantage of well-established architectures such as TL structures. As a result, it employs millions of images for classification procedure. To enable immediate implementation of such models, a trade-off between computing load and accuracy must be determined.
5. Additionally, the DL network should be trained with a large number of images to guarantee that the features are more generalized.

By employing a modified deep model and ensemble of learning based on the pool of SVM method with RBF kernel for disease detection in fruit leaves, the proposed study solved the identified research gaps. The following are some of the project's most significant contributions.

1. A novel and distinct DL architecture has been presented in this field of research. The first goal was to use color space to improve feature classification. The second stage uses TL to learn and enhance performance by learning distinct feature maps.
2. This is the first time a hybrid model based on the modified deep TL network and ensemble of learning has been used to diagnose disease in a significant number of fruit leaves, as far as we know.
3. Samples from a variety of approaches were used to train the design. We trained the architecture with 90000 and validated it with 10-fold cross-validation of images.

Section 2 focuses on fruits, citrus, and rice leaf diseases, along with related datasets and processing methods. Identifying fruits, citrus, and rice leaf diseases is the subject of Section 3, which examines several concepts and methodologies.  Section 4 tests the model described in this article and compares it to other models. There is a brief summary of the article in the Section 4.

## 2. Proposed Method

**Figure 1:**
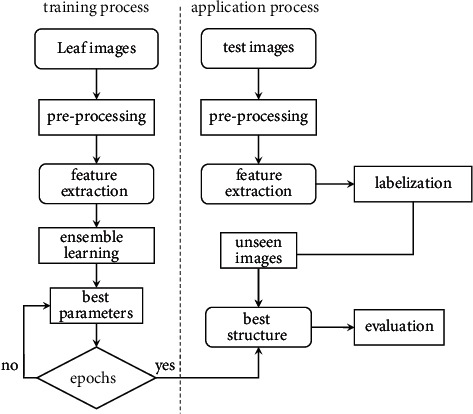
The general procedure for the suggested implementation approach is illustrated in this figure.

The suggested technique is depicted in Figure 1, which begins with feature extraction using a deep convolutional neural network model and ends with classification using an optimum support vector machine as the final decision layer.

### 2.1. Preprocessing

**Figure 2:**
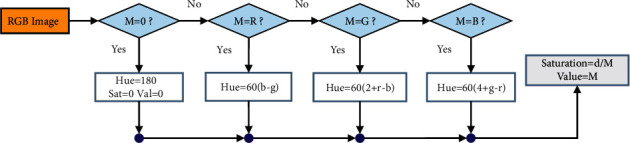
Graphic schematic from stages of RGB image to HSV image transform [40].

To accurately reproduce the appearance of colors in natural light on the image processing step, an HSV (color, saturation, and quantity) display must be used. Since the strongest color of HSV can be compared to white light, the terms “HSV” and “white light” are often used interchangeably (i.e., bright white light shining on a red object). In low light, a red object seems darker and brighter, while it appears redder and brighter in high light. We are in dire need of ensuring that no light is lost in the process; a single point source must be achieved in the light of leave images. A preprocessed RGB image is fed into the HSV converter to keep the brightness constant. The process of transforming an RGB image to an HSV image is shown visually in Figure 2.

### 2.2. Convolutional Neural Network (CNN)

We use nonlinearity and a series of convolutional filters to solve equation (1). There is a level of hierarchy in a CNN. The following layers (*x*_*j*_) are derived from the input signal *x* [41]:

(1) $$\begin{array}{c} x_{j}=\rho W_{j}x_{j}-1. \end{array}$$

However, even though *W*_*j*_ is a linear operator, this situation exhibits nonlinear behavior. Additionally, *W*_*j*_ is frequently employed in convolutions in CNNs, with *ρ* being either a rectifier max(*x*; 0) or an exponential sigmoid [1 + exp(–*x*)]^−1^. Convolutional filter stack *W*_*j*_ is assumed. As a result, the convolutions in each layer are defined as the total of the convolutions in the layer before it [41, 42].

(2) $$\begin{array}{c} x_{j}\left(u,k_{j}\right)=\rho \left(\sum_{k}\left(W_{j,k_{j}}\left(.,k\right)*x_{j-1}\left(.,k\right)\right)\left(u\right)\right). \end{array}$$

In addition, a discrete convolution process known as ^*∗*^ is employed [41]:

(3) $$\begin{array}{c} \left(g*f\right)\left(x\right)=\sum_{u=-\infty }^{\infty }g\left(u\right)f\left(x-u\right). \end{array}$$

CNN architecture optimization is a nonconvex issue. Weights *W*_*j*_ are frequently trained using stochastic gradient descent, with their gradients commonly determined using backpropagation. The reliance on data is a critical concern in deep learning. Deep learning models are largely reliant on massive volumes of data to be trained. In comparison to more standard machine learning algorithms, this is a significant improvement. The issue is that, in order to train the underlying data patterns, a large amount of data is required. TL can be used to address the issue of training data being distributed uniformly independent of the distribution of the test data (i.e., which motivates us to employ TL to address the problem of insufficient training data). VGG, DenseNet, and ResNet are three well-known deep learning networks that train their models using CNNs procedure.

### 2.3. Dilated ResNet

**Figure 3:**
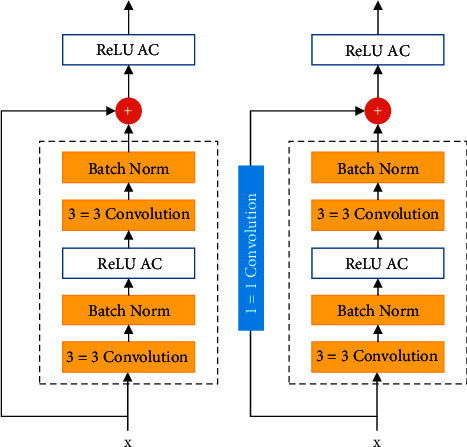
We use the ResNet residual block in the modified ResNet architecture: (a) direct mapping based on the training; (b) computation difference between the output and input.

ResNet's 3 × 3 Visual Geometry Group (VGG) full-layer design is the best on the market for transfer learning. The 3 × 3 output channels for each of the remaining convolutional layers are shown in Figure 3(a). After the data has been modified, it is submitted to ReLU layers. ReLU is activated following two convolutions. ResNet is based on the GoogLeNet structure as a starting point. Following the 7 × 7 layers are two strides and a convolution layer with up to 64, 3 × 3 input channels. It is feasible to connect the convolutional layer's outputs to a single input. Increasing the number of channels is one possibility. Furthermore, additional 1 × 1 convolution layers are also necessary. ResNet nodes are depicted in block form in Figure 3(b). ReLU takes nonlinear input into account. This layer has no effect on the channels preceding it. Due to the small volume of diseased spots in leaf images, the dilated ResNet structure aids in the detection of images with a small volume of disease. Due to the higher-quality leaf images in the ResNet architecture, a broader range of dilated structure-based features will be available. By employing samples and preceding layers, it is feasible to preserve fine-grained features. Thus, the lightweight structure of ResNet-18 was developed by experimentation with various convolution scales and dilation rates. Concatenation hence minimizes the computational cost while increasing the quantity of fine-grained attributes. Accordingly, samples must be collected in order to combine lower-level components into higher-level maps. The bottom and top layers should be merged to improve the identification of small volumes of illness in leafs.

**Figure 4:**
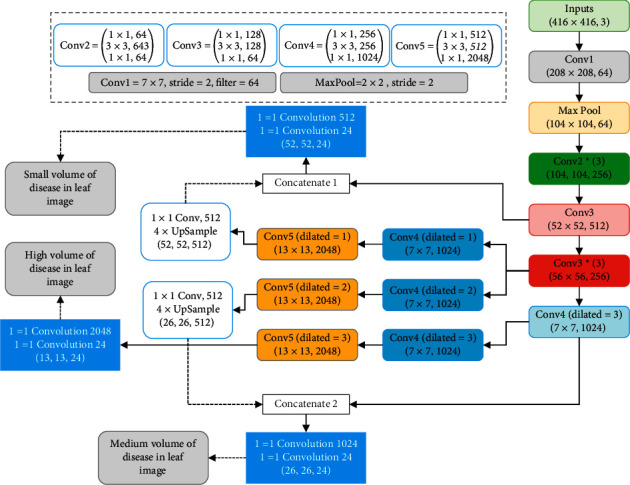
The overall architecture of modified transfer learning structure by using dilated and concatenation design.

Each of the 64 filters illustrated in Figure 4 has the 7 × 7 dimension, for a total of 64. To put it simply, *Conv*1 features a significantly larger 7 × 7 dimension, 64 filters, and a two-step stride. The stride length of the MaxPool 2 × 2 is two. Following feature extraction by deep structure, we further select the feature with the lowest computational complexity. The weights of features in a neighborhood can be computed using distance measures. It is feasible to minimize the size of the required feature vectors by employing the neighborhood component analysis (NCA) [43] technique, which stands for “supervised and nonparametric.” This approach allows for the enhancement and modification of the *k*-nearest neighbor (*k*-NN) structure. We choose this method to decrease the amount of feature vectors obtained from leaf images. This is because it assigns a positive weight to each feature and the feature rank can be computed using NCA. When a desired feature reduction strategy is applied, the NCA is used to estimate the feature weights. A feature set is subdivided into overlapping blocks. As a result, it is evident that *k* is a collection of smaller vectors.

### 2.4. Ensemble Learning

The classifying leaf images with the SVM model are a significant step toward generating the ideal hyperplane as a decision surface with the highest margin for interclasses of leaf diseases. As a result of the inherent separation issues, we present the radial basis function (RBF) kernel function and its associated decision components for SVM classification. A nonlinear kernel improves the overall performance of the SVM.

(4) $$\begin{array}{c} K\left(x,x_{i}\right)=\exp\left(-\frac{\gamma {\left\|x-x_{i}\right\|}^{2}}{2\sigma^{2}}\right). \end{array}$$

**Figure 5:**
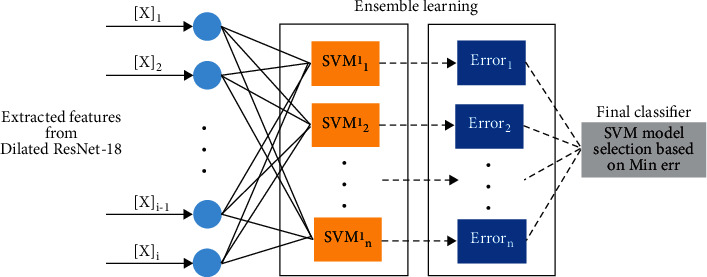
The procedure of decision-making part based on the ensemble of learning in pool of SVMs models with RBF kernel.

The determined RBF kernel in equation (4) contains fewer variables (*C* and *γ*), is mathematically simpler, and has fewer hyperparameters than other kernels. This is why it has gained widespread acceptance. The classification step utilizes training data to implement the SVM with RBF-based learning pool as the ensemble learning procedure. The extended ensemble learning structure is depicted in Figure 5 as a general schematic.

## 3. Experimental Results

This section describes the dataset and leaf image that were analyzed, as well as the model's results.

### 3.1. Procedure Setting

On Windows 10 PCs, the proposed solution was implemented using MATLAB R2021b. The simulation was conducted utilizing an Intel® Core™ i5-8500 processor (single CPU), 16 GB of RAM, and 16 GB of SSD RAM on a test computer. Additionally, we used additional applications, such as SPSS. The optimal parameters of the RBF kernel are then chosen for initialization. The initial ResNet-18 model had a primary learning rate (*µ*) of 0.002 and an epoch range of 100–1000. To avoid overfitting, the proposed ensemble learning system determined the ideal training iteration size by a mix of parameter tuning and early stopping.

### 3.2. Dataset

**Figure 6:**
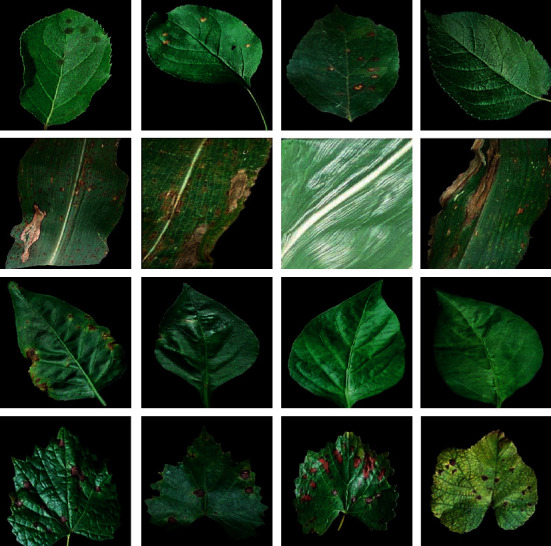
A collection of images of plant leaves, including the first row of apple fruit leaves, the second row of maize leaves, the third row of pepper leaves, and the fourth row of grape leaves, all of which have leaves with various pests. Additionally, some leaves are healthy.

**Figure 7:**
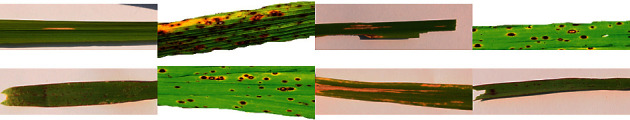
Rice leaves are divided into three different classes of bacteria, stains, and plants.

The images of plant leaves were created by analyzing six different types of plant leaves: apple fruit leaves, maize leaves, cotton plant leaves, grape fruit leaves, pepper leaves, and rice leaves. Figures 6 and 7 illustrate sample images, while Table 1 summarizes the number of images and other pertinent information.

### 3.3. Evaluations

Our early findings analyze how machine learning techniques based on ResNet structure families can be applied to improve leaf disease categorization. The feature extraction stage was carried out using an improved ResNet-18 (dResNet-18) version in ResNet structures with multiple classes and two classes, as indicated in Table 2. Additionally, a number of previously unknown leaf diseases were identified as a result of the multiclass classification based on region of interest (ROI) part and non-ROI segment (see Table 1).

While the remainder parts of the proposed method stay unchanged, the ResNet deep transfer learning family has been used in its place. Numerous parallels exist between the qualities associated with deep learning. To conduct the classification, the best SVM model is selected using an ensemble learning technique. However, the technique used to extract features by ResNet-101 and ResNet-164 is nearly identical.

While the proposed strategy for deep learning feature extraction outperformed other methods within the ResNet structure families in some circumstances, it is obvious that these methods occasionally produced a very small classification error.

**Figure 8:**
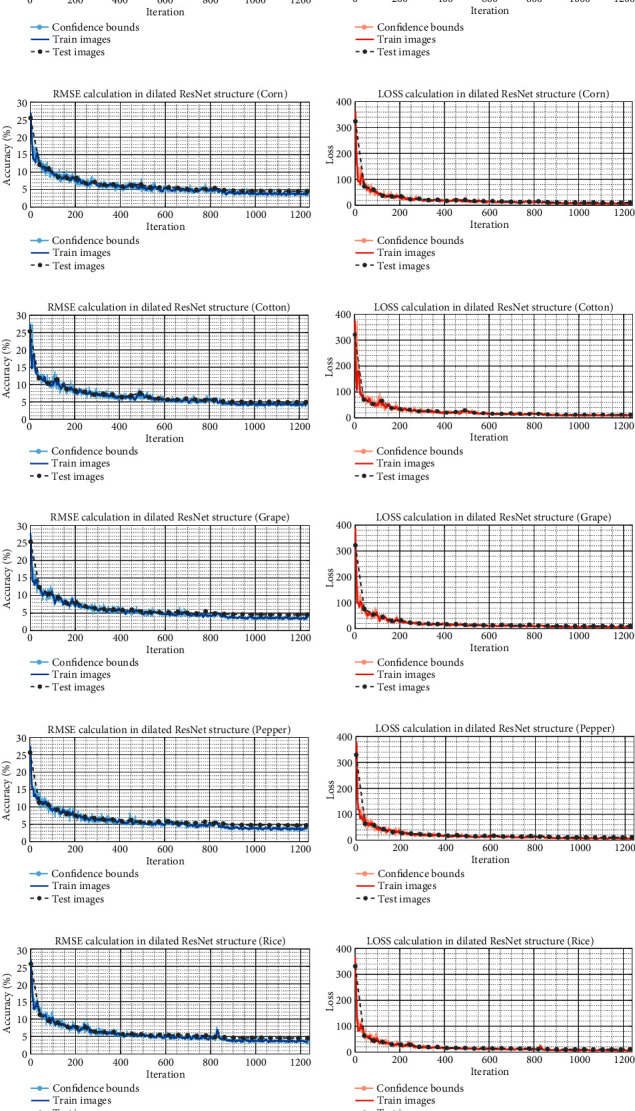
This figure depicts the RMSE convergence and loss function of the feature extraction section for six sets of acquired leaf images. (a, c, e, g, i, k) are the RMSE calculation and (b, d, f, h, j, l) are the loss computation for apple, corn, cotton, grape, pepper, and rice leaf images, respectively.

**Figure 9:**
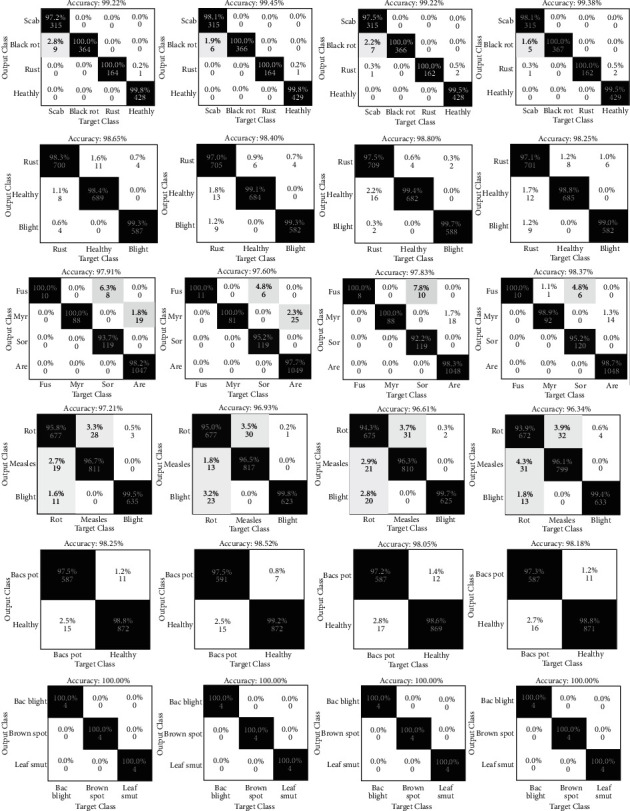
Confusion matrix: the suggested approach is used to diagnose leaf disease in a variety of hitherto unexplored datasets. The method's output is displayed four times at random. The first row represents apples, the second row represents corn, the third row represents flax, the fourth row represents grapes, the fifth row represents peppers, and the sixth row represents rice, accordingly.

Six different sets of gathered leaf photos are shown in Figure 8, each with its own RMSE convergence and loss function denoted by a curve and the lowest achievable value. The model's complexity is reduced by increasing the layer-wise reduction factor. As the model becomes simpler, the RMSE tends to rise. If the algorithm is trained to employ efficient features, it is feasible to improve discrimination outcomes. As a result, increasing the quantity of characteristics obtained by dilated deep transfer can help with learning. It is possible to create useful feature maps from a leaf image with only a few iterations and a short period of time. Because of the processing time involved, we may want to avoid deeper structures for real-time or near-real-time applications (particularly during training). The confusion matrices for the integrated model's categorization across all six datasets are shown in Figure 9.

While the classification accuracy of the grape and cotton leaf datasets is lower than that of other datasets, adding the proposed extracted features significantly improves classification accuracy. In addition, 10-fold cross-validation is used to demonstrate the ability of the system to classify leaf disease. For images of healthy or sick leaves, this approach asserts a 99.1% accuracy.

Using the approach explained here, numerous low and high intensities leaf diseases can be detected in a wide variety of fruits. It is reasonable to anticipate the proper classification rate because it consistently obtains a 99.1% accuracy rate in trials involving two classes in a range of situations. Our model achieves a precision score of 100% on each rice and apple leaves image. The proposed deep model and effective learning approaches for feature extraction may have resulted in appropriate sensitivity. The results show that the proposed method for diagnosing and classifying leaf diseases tends to be reliable. When a leaf image is complicated, as seen in Figure 9, various techniques' accuracy and precision rates often decrease (e.g., especially when the images are noisy or non-RoI image is selected as based for processing). The approach for analyzing leaf images, on the other hand, is consistent and reliable, making it a cost-effective method.

## 4. Discussion

This study employed a technique known as model engineering (ME) learning. There are various approaches to train a model for plant disease recognition; however, our model was trained using the dilated ResNet-18 framework for deep transfer learning.

**Figure 10:**
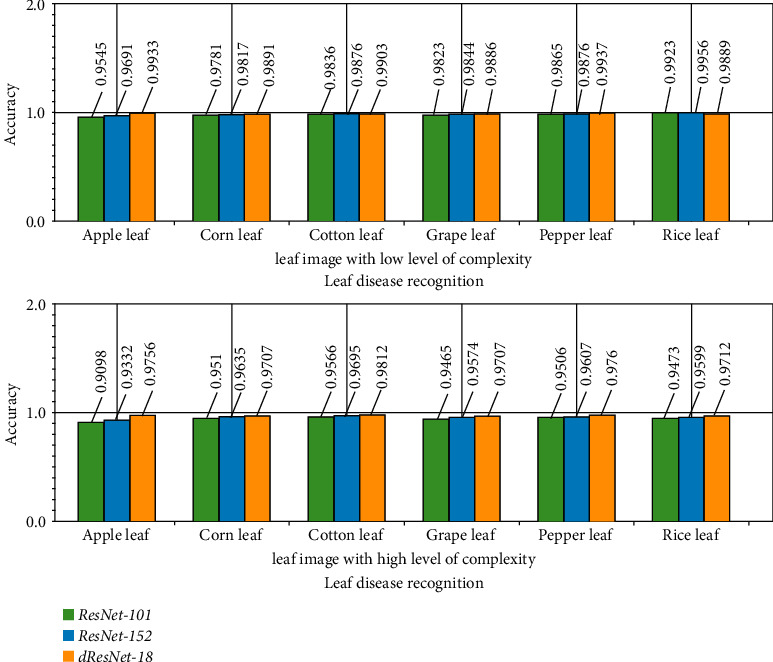
This figure illustrates the accuracy change for various TL model verification sets based on the low and high levels of complexity.

Our learning algorithms were all constructed using identical data and loss functions. The ME model described in this article was applied to a range of fruit leaf images. The RMSE and loss error change curves for each model's training set are displayed in Figure 8.  Figure 10 illustrates the accuracy change for various TL model verification sets based on the low and high levels of complexity.

The accuracy rates for the different decision-making part based on various test sets are shown in Table 3 based on *k*-nearest neighbor (*k*-NN), decision tree (DT), feedforward neural network (NN), SVM with linear kernel (SVM-L), SVM with RBF kernel (SVM-RBF), and Ensemble SVM-RBF (E-SVM-RBF).

The dilated learning model outperforms the typical ResNet-18 design in terms of stability. This model has an average accuracy of 98.5% on the test set for leaf disease recognition models. A test set accuracy of 97.93% is less than the suggested structure's accuracy of 97.93% in recognizing Grape or Cotton leaf diseases. For instance, our new deep learning model outperforms existing deep learning models by an average of 1 to 2 percentages. The addition of acceptable noise while learning a task aids in the improvement of models, which is analogous to removing unnecessary steps from the process. In contrast to conventional learning, interaction can assist in preventing the overfitting of different leaf image conditions from collapsing. As a result, the proposed ME learning technique can aid in the achievement of numerous, relatively related objectives. For all leaf disease recognition, the proper epochs were used to optimize the training time in the procedure. This resulted in a more rapid and constant drop in the recognition model's accuracy. Our ME model surpasses existing feature extraction-based deep TL models.

There are multiple distinct methods for incorporating pretrained structures into TL models. Minor changes are made using the TL approach. This was no longer essential as a result of the ensemble classification approach. Model reuse is distinguished by the fact that it uses the model's structure but not its pretrained parameters. We processed images of leaves on a variety of fruits using the approach indicated above. According to the results in Table 4, when using the DL structure with a limited number of epochs in the training stage for creating proper features with the appropriate metrics computation, the hybrid model is capable of recognizing leaf disease with a maximum accuracy of 99.1%.

Our model, which was taught to recognize leaves in a variety of fruits, demonstrated a reliable effect that might be used as actual smart technology in agriculture. Other studies looked into whether DL-based models are unique in their ability to distinguish between diseases from different time periods. The table below compares the dResNet-18 and other decision-making structures to a variety of feature extraction and ensemble learning models. To detect, recognize, and describe leaf disease in plants, many deep learning models are used. Various CNN models, such as S-CNN and F-CNN [17], EfficientNet [43], Hy-CNN [45], and the united deep learning model [46], have been applied to various plant leaf images by a number of authors. On plant leaves, a hybrid analytic model [47] obtains 95.1 percent precision, while other models achieve 92.01% precision [48]. The classification of images of coffee leaves is improved by texture image analysis [49]. In addition, our approach is a fast method that has less computational complexity than other similar methods in leaf disease classification.

As a result of its many advantages, deep learning in data science can lead to more efficient processing models [53]. A decision-making approach proposed in this study could be of significant help in classifying leaf diseases. By using deep learning, accuracy can be continuously increased and knowledge can be continuously gained without supervision. Additionally, data analysts will benefit from more accurate and concise results as a result of this initiative.

## 5. Conclusion

This article analyzes and provides a dataset of six different types of crop disease leaves. The deep CNN-based dilated ResNet-18 model is trained and evaluated using data processing, preprocessing of the dataset, training, and experimental procedure. The proposed model is constructed and tested to determine whether it can improve on the outcomes obtained thus far. Various datasets indicate that the metrics parameters are stronger and higher than those of other techniques. As a result, our proposed research work increased the accuracy of various leaf images of various fruits by 99.1 percent, including Apple, Corn, Cotton, Grape, Pepper, and Rice. We were able to achieve the highest level of performance possible with our model when it came to on-field crops, leaf images, and disease classification and analysis. Agriculture and food production are the core objectives of university and government-funded research. It is intended that real datasets comprising a variety of leaf crops would be collected and processed for usage in deep learning models in the future. Crop images will significantly benefit from the future usage of hybrid models based on improved TL architecture and traditional models. Farmers gain from our efforts because we encourage and stimulate agriculture, which boosts agricultural revenue and contributes to the development of strong countries. Additionally, in the future, the authors of this study will attempt to reduce the algorithm's computational complexity and execution time.

## Figures and Tables

**Table 1:** This table shows the details of the leaves of the various fruits analyzed in the study.

Leaf type	Number of classes	Disease type	Dataset size	Sample		
Apple	4	Scab	3150	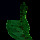	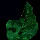	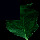
		Black rot	3726	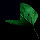	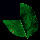	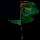
		Cedar apple rust	1650	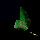	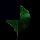	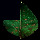
		Healthy	4284	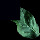	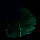	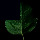

Corn	3	Common rust	7152	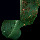	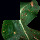	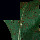
		Healthy	6972	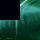	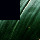	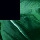
		Northern leaf blight	5910	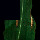	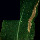	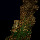

Cotton	4	*Fusarium* wilt	174	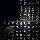	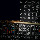	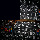
		Myrothecium leaf spot	1062	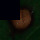	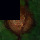	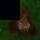
		Mela (soreshin)	1188	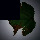	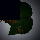	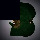
		Areolate mildew	10482	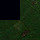	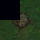	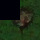

Grape	3	Black rot	7080	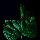	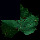	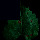
		Black measles	8304	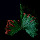	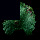	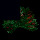
		Leaf blight	6456	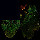	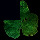	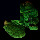

Pepper	2	Bacterial spot	5982	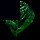	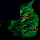	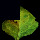
		Healthy	8868	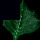	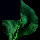	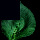

Rice	3	Bacterial leaf blight	40	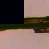	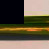	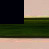
		Brown spot	40	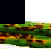	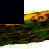	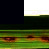
		Leaf smut	40	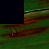	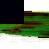	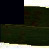

**Table 2:** Comparison of the findings of ResNet family's error in leaf disease classification using normal and ROI images for a multiclass problem.

Leaf type	ResNet-164		ResNet-152		ResNet-101		ResNet-50		ResNet-18		dResNet-18	
	Min	Max	Min	Max	Min	Max	Min	Max	Min	Max	Min	Max
Apple	0.03	0.07	0.04	0.08	0.04	0.09	0.05	0.08	0.06	0.10	**0.01**	**0.03**
Corn	0.06	0.12	0.06	0.12	0.07	0.11	0.07	0.12	0.09	0.13	**0.03**	**0.06**
Cotton	0.03	0.08	0.03	0.09	0.04	0.09	0.04	0.09	0.05	0.10	**0.02**	**0.05**
Grape	**0.04**	**0.06**	0.06	0.08	0.07	0.09	0.07	0.11	0.07	0.12	0.04	0.08
Pepper	0.03	0.06	0.04	0.07	0.04	0.07	0.04	0.08	0.05	0.08	**0.02**	**0.04**
Rice	0.005	0.01	0.01	0.015	0.008	0.02	0.01	0.02	0.01	0.03	**0.002**	**0.008**
Apple (ROI)	0.02	0.06	0.03	0.05	0.03	0.05	0.03	0.06	0.04	0.07	**0.007**	**0.02**
Corn (ROI)	0.04	0.08	0.04	0.09	0.05	0.09	0.06	0.10	0.07	0.10	**0.01**	**0.04**
Cotton (ROI)	0.01	0.06	0.02	0.07	0.02	0.08	0.03	0.08	0.03	0.09	**0.016**	**0.03**
Grape (ROI)	**0.01**	**0.05**	0.03	0.07	0.04	0.07	0.04	0.09	0.06	0.10	0.013	0.05
Pepper (ROI)	0.02	0.05	0.03	0.06	0.03	0.07	0.03	0.07	0.03	0.08	**0.009**	**0.04**
Rice (ROI)	0.004	0.008	0.005	0.018	0.006	0.02	0.008	0.02	0.009	0.02	**0.001**	**0.007**
**Average**	0.036	0.086	0.047	0.097	0.053	0.10	0.055	0.11	0.068	0.12	**0.009**	**0.044**

**Table 3:** The comparison of the different decision-making part based on various test sets.

Leaf image type	*k*-NN (%)	DT (%)	NN (%)	SVM-L (%)	SVM-RBF (%)	E-SVM-RBF (%)
Apple	97.56	97.31	96.11	95.48	98.13	99.47
Corn	97.08	96.83	95.78	94.11	97.29	98.69
Cotton	95.71	96.10	95.39	96.73	97.76	98.91
Grape	96.18	96.51	96.26	95.31	97.07	98.74
Pepper	97.09	96.65	95.62	96.13	97.87	99.12
Rice	98.31	98.87	97.91	96.44	98.76	100

**Table 4:** Comparison of proposed method with other deep and classical learning structures.

Reference	Type of leaf	Model	Accuracy (%)	Computational complexity
Chen et al. [10]	Rice	VGGNet	92.00	High
Sharma et al. [25]	Tomato	S-CNN and F-CNN	98.30	High
Atila et al. [44]	Plant leaf	EfficientNet	96.18	High
Kaur et al. [45]	Grape	Hy-CNN	98.70	Medium
Ji et al. [46]	Grape	United model	98.20	High
Gadekallu et al. [47]	Plant leaf	Whale and DL	95.10	Medium
Azimi et al. [48]	Crop	FCNN and SCNN	92.01	High
Joshi et al. [49]	Coffee	Deep CNN	98.00	High
Paymode and Malode [50]	Grape and tomato	VGG	98.40 and 95.71	High
Jiang et al. [51]	Rice and wheat	VGG16, DenseNet-121, and ResNet-50	97.22 and 98.75	High
Nandhini and Ashokkumar [52]	Plant leaf	DenseNet-121 and optimization algorithm	98.70	High
**Our model**	Plant leaf	Dilated TL and ensemble learning	99.10	Medium

## Data Availability

All the data and codes are available from corresponding authors.
